# The Role of Imaging Modalities in Estimating Myocardial Viability: A Narrative Review

**DOI:** 10.3390/jcm14155529

**Published:** 2025-08-06

**Authors:** Vishakha Modak, Vikyath Satish, Maisha Maliha, Sriram S. Kumar, Panagiota Christia

**Affiliations:** 1 Jacobi Medical Center, Albert Einstein College of Medicine, New York, NY 10461, USA; satishv@nychhc.org (V.S.); maliham@nychhc.org (M.M.); sunilkus@nychhc.org (S.S.K.); 2 Department of Medicine (Cardiology) and Radiology, NYU Grossman School of Medicine, New York, NY 10016, USA; panagiota.christia@nyulangone.org

**Keywords:** myocardial viability, imaging modalities, imaging

## Abstract

Myocardial viability assessment plays a critical role in the clinical management of patients with ischemic heart disease, particularly in guiding revascularization decisions. Various non-invasive imaging modalities have been developed and refined to evaluate viable myocardium, each offering unique insights into myocardial perfusion, metabolism, and contractile function. This review examines the comparative strengths and limitations of key imaging techniques. Understanding the pathophysiological basis and diagnostic capabilities of these modalities enables clinicians to tailor viability assessments to individual patient profiles, ultimately enhancing decision-making and optimizing outcomes in ischemic cardiomyopathy.

## 1. Introduction

Ischemic heart disease remains the leading cause of cardiovascular mortality in the United States and the predominant contributor to heart failure in developed nations [1]. According to the American Heart Association, approximately 50% of heart failure cases are attributed to ischemic injury, leading to functional myocardial impairment. The extent of dysfunction largely depends on the severity and duration of the ischemic insult. In cases where ischemia is transient or less severe, myocardial function may be temporarily depressed but remains potentially reversible—a phenomenon known as myocardial viability. Early identification of salvageable myocardium is therefore critical, as it can guide revascularization strategies that improve myocardial function and, in turn, enhance overall clinical outcomes. Over the past few decades, various imaging modalities have been developed to detect viable myocardium. These modalities differ significantly in sensitivity, specificity, cost, and clinical applicability. Therefore, selecting the most appropriate imaging modality requires careful consideration of institutional resources and patient-specific factors such as comorbidities, contraindications, and clinical stability. This review aims to explore the imaging techniques available for assessing myocardial viability while providing a practical framework to support modality selection across various clinical settings.

### 1.1. Viable Myocardium: Myocardial Stunning and Myocardial Hibernation

Under the broader concept of myocardial viability, two clinically important phenomena should be distinguished: myocardial stunning and myocardial hibernation. The term myocardial stunning was introduced in the 1980s and refers to prolonged post-ischemic ventricular dysfunction that occurs after brief periods of ischemia [2]. Foundational studies indicated that myocardial cells required approximately one week to restore structural normalcy and recover baseline ATP levels [3]. In clinical practice, myocardial stunning is observed in various contexts involving acute ischemia, commonly seen in patients undergoing percutaneous coronary intervention (PCI) or thrombolytic therapy for acute myocardial infarction, where transient left ventricular systolic function occurs post-reperfusion. Research has shown that patients undergoing elective PCI may demonstrate temporary systolic dysfunction following balloon inflation during angioplasty [4]. Similar patterns of transient dysfunction have also been documented following cardiac surgery, with full recovery of contractile function occurring over time [5]. In contrast, myocardial hibernation refers to chronic yet reversible ventricular dysfunction resulting from sustained ischemia. This condition persists until blood flow is restored, typically via coronary revascularization [6]. Hibernation is considered an adaptive survival mechanism that allows myocardial cells to endure chronic underperfusion by reducing their energy demand and altering metabolic processes. Specifically, hibernating myocardium exhibits a metabolic shift from fatty acid oxidation to glucose metabolism, which is more oxygen efficient and better suited to limited perfusion [7]. This state is often associated with chronic coronary artery disease and particularly with patients with multivessel disease, where sustained hypoperfusion leads to a reduction in myocardial contractility. Unlike stunning, which arises from acute ischemia and resolves spontaneously, hibernation persists and is reversed only after restoration of perfusion.

When ischemia becomes prolonged and severe, it progresses to irreversible myocardial injury and necrosis. During this process, ATP stores are depleted, cellular homeostasis is lost, and mitochondrial function deteriorates, leading to cell death. The resulting necrosis triggers the release of intracellular enzymes, activates inflammatory pathways, and ultimately culminates in the formation of non-contractile fibrous tissue (scar). This scarred myocardium lacks the potential for functional recovery even after revascularization, unlike stunned or hibernating tissue.

Accurate differentiation between viable and non-viable myocardium is essential when evaluating patients with left ventricular systolic dysfunction and planning revascularization strategies. Several imaging modalities help in this determination. Early identification and revascularization of viable myocardium have been associated with improved survival and better functional outcomes in patients with ischemic cardiomyopathy [8].

### 1.2. Imaging Techniques for Myocardial Viability

Various imaging modalities are available to evaluate myocardial viability, each providing unique insights into the biochemical, metabolic, and structural characteristics that differentiate viable from non-viable myocardium. These techniques play a pivotal role in determining the likelihood of functional recovery following revascularization.

Commonly used imaging techniques for assessing myocardial viability include echocardiography, magnetic resonance imaging (MRI), positron emission tomography (PET), single-photon emission computed tomography (SPECT), and computed tomography (CT). Each modality relies on distinct physiological principles—ranging from myocardial perfusion and wall motion analysis to metabolic activity and delayed contrast enhancement—to evaluate tissue viability. In the following sections of this review, we will examine these imaging techniques in detail, highlighting their respective advantages, limitations, and clinical applications in the management of ischemic cardiomyopathy.

## 2. Echocardiography

### 2.1. Dobutamine Stress Echocardiography

This modality identifies structural changes that differentiate viable tissue from necrotic tissue. In myocardial tissue with impaired contraction at rest, low doses of dobutamine (2.5–10 µg·kg^−1^·min^−1^) elicit augmented contractility in hibernating myocardium, reflecting preserved contractile reserve [9]. However, at progressively higher doses (10–40 µg·kg^−1^·min^−1^), the hibernating segment may develop abnormal wall motion as compromised coronary blood flow becomes insufficient to meet the increased metabolic demands. This characteristic pattern, called the “biphasic response,” is a key echocardiographic marker of myocardial viability [10]. It is a predictive indicator of myocardial viability after revascularization, outperforming other patterns such as uniphasic worsening or sustained improvement in wall motion [11]. (refer to Table 1)

One of the main advantages of dobutamine stress echocardiography is its ability to assess non-invasively contractile reserve with high specificity. It is also widely available, does not require ionizing radiation, and is inexpensive. A study found that the biphasic response to dobutamine strongly predicts regional functional recovery after revascularization, outperforming several other imaging modalities [9]. Additionally, clinical evidence suggests that four or more segments exhibiting a biphasic response on dobutamine echocardiography correlate with better post-procedure survival rates and functional improvement [10]. This emphasizes the prognostic value of DSE in clinical decision-making, particularly in patients being evaluated for coronary artery bypass grafting or percutaneous interventions.

Despite its strengths, dobutamine stress echocardiogram (DSE) is not without limitations. It is highly operator-dependent, meaning image acquisition and interpretation can vary significantly depending on the sonographer’s and physician’s expertise. The quality of the images and the accuracy of the test can vary based on the technician’s skill and experience. Additionally, suboptimal acoustic windows can degrade image quality, such as in patients with obesity, chest deformities, breast implants, or chronic obstructive pulmonary disease (COPD), limiting diagnostic accuracy [11]. The test can also be challenging in heart failure patients, as wall motion abnormalities may be diffuse, making it challenging to distinguish viable from nonviable myocardium. Pre-existing arrhythmias, such as atrial fibrillation, frequent premature beats, or ventricular tachycardia, can also interfere with the test by mimicking ischemic wall motion changes or increasing the risk of arrhythmia during stress [12]. Furthermore, DSE is contraindicated in patients with severe aortic stenosis, unstable angina, or those who have had a recent myocardial infarction, as the induced stress may worsen these conditions or lead to adverse cardiac events under pharmacologic stress.

### 2.2. Contrast Enhanced Echocardiography (CEE)

Contrast-enhanced echocardiography (CEE) uses microbubble contrast agents to assess myocardial perfusion, offering insight into tissue viability based on capillary blood flow [13]. When injected intravenously, these microbubbles enhance endocardial border delineation and enable real-time perfusion imaging. During imaging, a high mechanical index (MI) burst destroyed the microbubbles, and the replenishment rate was observed. Rapid replenishment (<5 s) suggests viable myocardium, while persistent absence of opacification (>10 s) indicates nonviable tissue [14]. CEE has demonstrated strong performance in quantifying infarct size and correlating perfusion with functional recovery after revascularization [15]. However, its use in viability assessment remains limited. It has not been FDA-approved for this indication and is hindered by high inter-observer variability and the need for specialized training and imaging artifacts. While useful as an adjunct to DSE, especially in technically complex cases, CEE is not yet a first line for assessing viability.

### 2.3. Myocardial Strain Imaging with Speckle Tracking Echocardiography

Speckle tracking echocardiography (STE) evaluates regional myocardial deformation, offering a detailed assessment of contractile function. Strain values are measured from end-systole to end-diastole, and more negative values indicate better function. A reduction in strain magnitude can suggest dysfunctional or infarcted tissue. A longitudinal strain cutoff of −13.7% has been identified as predictive of recovery post-revascularization [16]. STE provides objective quantitative data with high temporal resolution and helps detect subtle dysfunction. Combined with dobutamine echocardiography, strain imaging may enhance sensitivity in detecting viable myocardium [17]. However, like CEE, its application is limited by availability, operator expertise, and image quality. In routine clinical practice, it is more commonly used as a complementary tool.

## 3. Cardiac Magnetic Resonance Imaging (CMR)

Cardiac magnetic resonance (CMR) is a powerful diagnostic tool that evaluates myocardial viability by analyzing parameters such as scar tissue burden, wall thickness, and contractile function. It employs gadolinium enhancement to distinguish between infarcted and viable tissue. Understanding the pathophysiology of infarcted tissue is crucial; acutely infarcted tissue shows a loss of membrane integrity, leading to increased interstitial space. Gadolinium, which cannot penetrate intact cell membranes and is metabolically inert, accumulates in areas with compromised cellular integrity. In contrast, chronically infarcted tissue displays increased interstitial space due to extracellular fibrosis instead of acute cell membrane disruption. In both acute and chronic infarctions, elevated extracellular gadolinium results in a hyper-enhanced signal on T1 imaging, effectively identifying infarcted areas [18]. First-pass perfusion imaging in CMR assesses myocardial blood flow and identifies ischemic but viable myocardium prior to performing late gadolinium enhancement (LGE). In this technique, a contrast agent is injected, and its uptake by the myocardium is monitored. Reduced uptake (a perfusion defect) signals ischemic myocardium that remains viable and holds potential for recovery post-revascularization.

### 3.1. Quantification of Transmural Infarction to Determine Viability

Late gadolinium enhancement (LGE) on CMR quantifies the transmural extent of infarction, providing prognostic information on the likelihood of functional recovery after a myocardial infarction (MI). Complete (100%) transmural enhancement indicates full infarction with very low chances of recovery, as the tissue is deemed non-viable. Subendocardial enhancement (less than 50% thickness) indicates partially viable myocardium with a promising recovery potential after revascularization. Conversely, involvement exceeding 50% of myocardial thickness usually points to non-viable tissue with minimal recovery chances. Research by Choi et al. demonstrated that greater transmural infarction extent correlates with reduced likelihood of functional improvement [19]. The chances of contractile recovery post-MI are significantly higher when infarction is limited to less than 25%, illustrating that smaller infarctions are more likely to regain contractile function.

### 3.2. Role of CMR in Quantifying Wall Thickness to Assess Viability

CMR is essential for precise wall thickness measurements to evaluate myocardial viability. However, despite providing detailed data, it has limited specificity (56%) for predicting functional recovery post-revascularization when end-diastolic wall thickness exceeds 5.5 mm. A study by Shah et al. highlighted that scar tissue burden is a more accurate predictor of recovery than wall thinning [20]. Additionally, quantitative assessments of dobutamine-induced systolic wall thickening in chronic infarcts reliably forecast left ventricular (LV) functional recovery, with sensitivity and specificity rates of 89% and 94%, respectively [19].

### 3.3. Advancements in AI-Enhanced MRI for Myocardial Viability Assessment

Research by Ghaffari Jolfay et al. has identified the efficacy of strain imaging and T1/T2 mapping on MRI for segmental viability assessment, moving beyond traditional contrast methods. The study revealed that T1/T2 mapping excels in predicting tissue viability in regions supplied by the right coronary artery, while circumferential strain analysis is more effective in areas supplied by the left coronary artery. This suggests potential for viability assessment based on perfusion patterns and introduces the possibility of integrating machine learning algorithms into viability analysis [21].

Cardiac MRI (CMR) boasts the highest sensitivity (95%) in viability assessment compared to alternative techniques. It can evaluate multiple parameters in a single scan, including wall thickness, volumes, ejection fraction, myocardial function, scar tissue, edema, and contractile reserve. When paired with dobutamine stress (stress CMR), this method further examines the myocardium’s contractile reserve, akin to dobutamine echocardiography, but offers superior spatial resolution. The primary limitations of CMR include its high cost and restricted availability. While chronic kidney disease and the presence of implantable devices, such as pacemakers, are no longer considered absolute contraindications, they may still pose challenges depending on individual cases. Moreover, patients experiencing claustrophobia may find it difficult to tolerate the MRI scanner’s confined space, which can further restrict modality use.

**Figure 1:**
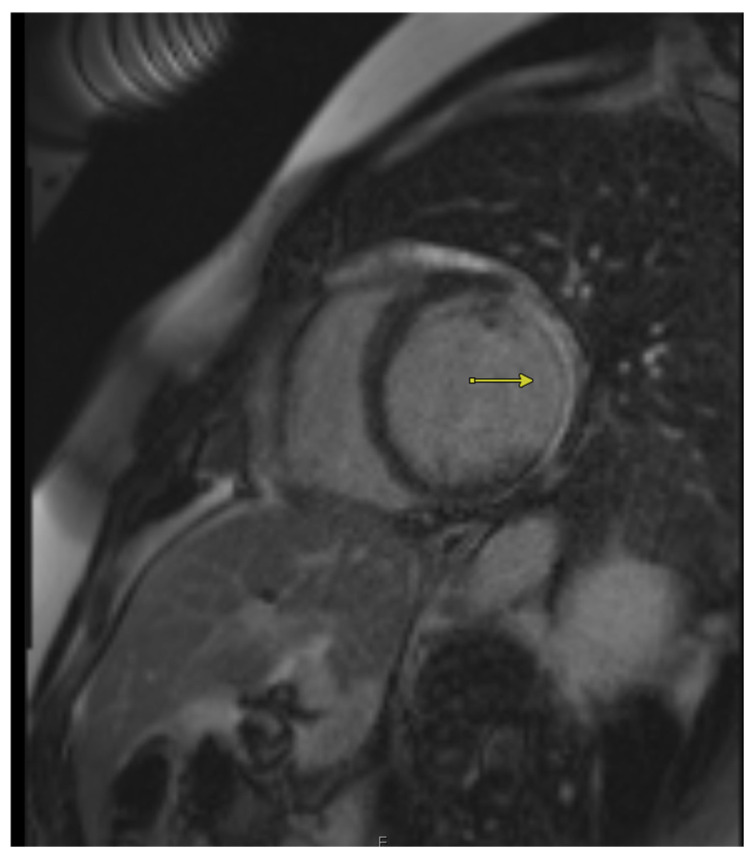
Cardiac MRI showing late gadolinium enhancement in a patient with chronic ischemic cardiomyopathy. Areas of enhancement (arrows) indicate non-viable myocardium.

## 4. Positron Emission Tomography (PET Scan)

Myocardial cells mainly generate energy from fatty acids; however, in cases of hibernating myocardium, the heart shifts its primary energy source to glucose to optimize ATP production. This metabolic adaptation forms the basis of FDG PET (fluorodeoxyglucose) scans for identifying viable cardiac tissue. The FDG PET scan involves a two-step process that begins with resting myocardial perfusion imaging to evaluate blood flow to the heart. Typically, PET imaging uses two tracers: 18F-FDG for glucose metabolism, and a perfusion tracer such as Rubidium-82, Oxygen-15 water, or Nitrogen-13 ammonia [22]. Normal and stunned myocardium show normal perfusion and glucose metabolism, while hibernating myocardium displays reduced perfusion coupled with heightened glucose metabolism [23]. Non-viable tissue typically reflects absent or significantly reduced perfusion and glucose metabolism. A notable advantage of FDG PET is its lower radiation exposure compared to SPECT. Furthermore, it allows for a more detailed evaluation, combining insights on both perfusion and metabolic activity. Nevertheless, the standard FDG PET protocol is hampered by a lengthy metabolic preparation process involving oral glucose followed by intravenous (IV) insulin to boost myocardial tracer uptake. Recent research by Mhalanga et al. proposed a revised protocol employing IV glucose alongside IV insulin loading, decreasing metabolic preparation time to 51 min—a 61% reduction from earlier methods [24]. Despite its utility, PET imaging remains costly and less accessible, particularly with specific tracers like ammonia, requiring an on-site cyclotron for production.

**Figure 2:**
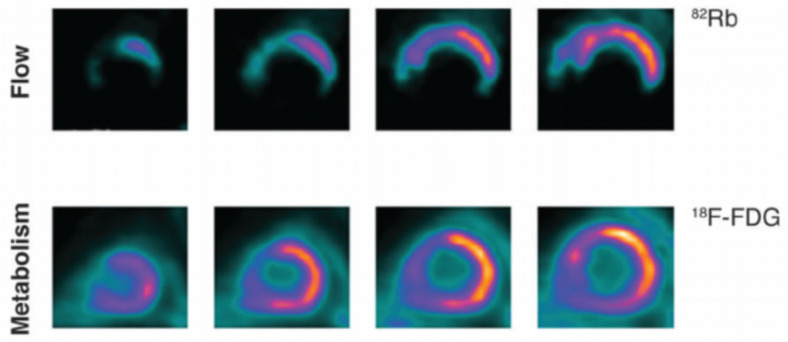
FDG-PET imaging demonstrating perfusion–metabolism mismatch—reduced perfusion (**top row**) with preserved glucose uptake (**bottom row**)—consistent with viable but hibernating myocardium. Adapted from Osterholt et al., “Targeted Metabolic Imaging to Improve the Management of Heart Disease,” under open-access terms [25].

## 5. Single Photon Emission Computed Tomography (SPECT)

Single-photon emission computed tomography (SPECT) is a nuclear imaging modality that utilizes tracers like thallium-201 (201 Tl) and technetium (99mTc) to evaluate myocardial viability. It operates on the principle of assessing cell membrane integrity, a key indicator of myocardial viability. Viable tissue maintains intact cell membranes, facilitating thallium uptake through active transport. This differs from non-viable, necrotic tissue with disrupted membranes that fail to absorb the tracer, resulting in decreased or absent uptake. Tracer uptake of more than 50% indicates viable tissue with the potential for functional recovery after revascularization [26]. These tracers are comparable in their ability to differentiate viable from non-viable tissue and predict functional recovery following revascularization. However, a distinctive feature of 201 Tl is its redistribution phenomenon, where the tracer gradually re-enters viable tissue over time. After initial uptake, the tracer gradually reenters viable but initially underperfused tissue over time. This allows for a rest stress redistribution imaging protocol, where delayed imaging, typically performed four hours after the initial injection, can reveal additional viable tissue that was not apparent during the initial scan [27]. In some cases, repeat imaging extending up to 24 h with or without reinjection of thallium can improve diagnostic sensitivity. This redistribution property enhances the ability to detect hibernating myocardium but also contributes to the test’s main limitation: it is time-consuming. It requires a prolonged imaging protocol, which can be burdensome for patients and staff.

Common technetium-based 99mTc-radiotracers such as sestamibi and tetrofosmin have gained popularity for myocardial viability assessment due to their shorter imaging protocols and superior image quality. The uptake of these tracers in myocardial cells is dependent on mitochondrial membrane integrity. Nitrates administered alongside technetium tracers enhance tracer uptake by improving perfusion and tracer delivery to the hibernating tissue [28]. Unlike 201 Tl, technetium-based tracers do not exhibit redistribution, leading to a shorter protocol duration for viability detection. Additional benefits include lower radiation exposure and superior spatial resolution compared to thallium-based imaging, making these agents more favorable in clinical settings with limited resources or time constraints.

Despite its advantages, SPECT has some limitations. While it demonstrates high sensitivity for detecting viable myocardium, it suffers from relatively low specificity, which can lead to false positives and overestimating viability. This may lead to unnecessary revascularization procedures. The spatial resolution of SPECT is inferior to other imaging modalities like PET or cardiac MRI, limiting its ability to localize small regions of viable myocardium precisely. Furthermore, SPECT involves a high radiation dose, particularly with thallium protocols, raising concerns for patients requiring multiple imaging studies or those at higher risk from cumulative radiation exposure.

In summary, SPECT remains a valuable tool for assessing myocardial viability, particularly when advanced imaging modalities are unavailable. Its high sensitivity aids in detecting viable tissue; however, limitations such as low specificity, high radiation exposure, and poor spatial resolution must be carefully considered when interpreting results and planning revascularization strategies.

**Figure 3:**
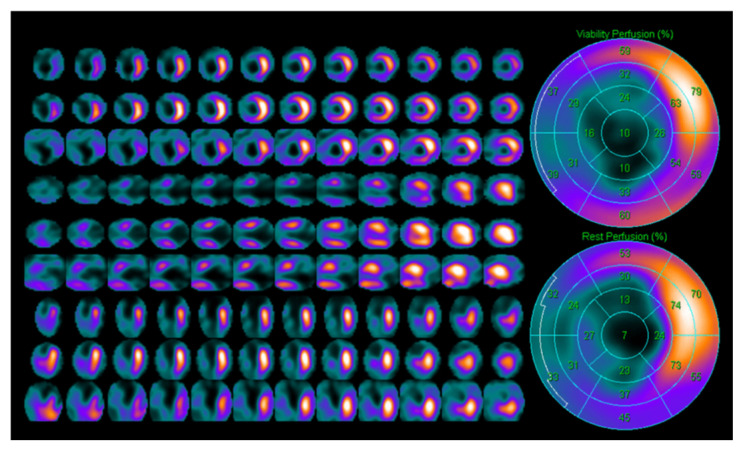
Rest–redistribution Thallium-201 SPECT imaging showing delayed uptake in the inferior wall, indicative of viable myocardium post-infarction. Adapted from Santoyo-Saavedra et al., 2022, under open-access terms [29].

## 6. Computer Tomography Scan (CT SCAN)

Multi-slice CT (MSCT) is an emerging technique for assessing myocardial viability, with delayed enhancement CT imaging showing comparable results to late gadolinium enhancement cardiac MRI (LGE-CMR). The principle of this modality mirrors that of LGE CMR: iodinated contrast has kinetics similar to chelated gadolinium and accumulates in the extracellular space. Infarcted myocardium, characterized by increased extracellular volume due to cell death and fibrosis, retains contrast longer and appears hyper-enhanced on late-phase CT images.

However, no standardized protocol exists for the timing of image acquisition after contrast administration. Studies differ on the optimal timing, ranging from five to 15 min [30,31]. Jacquier et al. found a better correlation with MRI when imaging was performed five minutes after contrast administration due to improved noise-to-signal ratios [31]. In contrast, Baks et al. reported improved alignment with MRI findings when imaging occurred 15 min post-injection. These discrepancies underscore the need for further studies to establish optimal imaging timing for viability assessment with MSCT [32].

MSCT has the advantage of being widely available compared to PET and is often more accessible than MRI. It also has high spatial resolution and faster acquisition time compared to cardiac MRI, which is beneficial for acutely ill patients or those unable to tolerate longer imaging sessions due to claustrophobia. Despite these strengths, MSCT has limitations, including contrast-induced nephropathy, allergic contrast reactions, and radiation exposure. To mitigate these concerns, newer protocols recommend using low kilovoltage settings of 80 kV, which can reduce the contrast burden by more than 50% compared to standard protocols [33]. As technology evolves, MSCT may become a more prominent modality in the non-invasive assessment of viability, particularly in institutions without access to PET.

CT Myocardial Perfusion Imaging:

Coronary CT Angiography (CCTA) is a valuable non-invasive imaging modality for the detection of coronary artery disease (CAD). However, a major limitation of CCTA is its inability to provide information about the functional status of the affected myocardial tissue. To address this, an advanced and emerging technique is the myocardial perfusion imaging (CT MPI), which allows for an assessment of myocardial perfusion and viability. CT MPI consists of two phases: rest and stress imaging [34]. Rest imaging is typically like CCTA and helps estimate the location and severity of obstructive lesions. Stress imaging, on the other hand, uses a pharmacologic stressor—commonly adenosine—to induce coronary vasodilation, simulating exercise conditions. The myocardial response to the stressor is then evaluated to identify perfusion defects. By acquiring images at multiple time points following iodine contrast injection, attenuation curves are generated, offering a quantitative assessment of myocardial perfusion over time. The protocol selection for CT MPI depends on the risk profile of the coronary lesions seen on CCTA. In patients with high-risk lesions (e.g., severe stenosis or high-risk plaque features), a stress-first protocol is used to promptly assess for functionally significant ischemia. In contrast, for low to intermediate-risk lesions, a rest-first protocol is generally preferred, allowing baseline evaluation before proceeding to stress imaging if necessary. When combined with CCTA, CT MPI significantly enhances diagnostic yield by integrating both anatomic and functional information. This hybrid approach has shown diagnostic performance comparable to highly sensitive modalities such as PET and cardiac MRI in detecting viable and ischemic myocardium. Despite its promising potential, CT MPI faces several limitations. These include higher radiation exposure, the requirement for advanced imaging systems that are not widely available, and a lack of standardization in imaging protocols and interpretation. Continued technological advancements and consensus on standardized protocols are needed to support wider clinical adoption of this powerful diagnostic tool [35].

## 7. Discussion

The concept of viable myocardium has evolved over the past three decades due to its potential implications for guiding revascularization therapy. Viable myocardium refers to hypo contractile or akinetic heart tissue that remains metabolically active and is believed to regain function with revascularization. Numerous meta-analyses and retrospective studies have supported this hypothesis, suggesting that restoring perfusion in viable tissue may lead to improved systolic function, reduced mortality, and enhanced quality of life. A pivotal meta-analysis by Allman et al. demonstrated that patients with viable myocardial tissue who underwent revascularization therapy experienced nearly an 80% reduction in yearly mortality rates compared to those receiving medical treatment alone [36]. While compelling, these retrospective findings have not yet been replicated in prospective randomized trials.

Key randomized trials, including the STICH (Surgical Treatment for Ischemic Heart Failure) trial, have investigated the survival benefit of revascularization in patients with viable myocardium. STICH did not demonstrate a survival benefit with revascularization compared to medical therapy alone, despite improved ejection fraction (EF) in both subgroups [37,38]. However, interpretation of these results is limited by the viability assessment tools employed, namely, dobutamine stress echocardiography and SPECT, which are now recognized as having lower diagnostic accuracy than advanced modalities such as late gadolinium enhancement MRI and FDG PET. Likewise, the REVIVED-BCIS2 trial (Revascularization for Ischemic Ventricular Dysfunction) also failed to demonstrate a survival benefit of PCI-based revascularization compared to medical therapy alone, reinforcing the findings of the STICH trial [39]. However, it revealed that the extent of non-viable myocardium was associated with worse outcomes, underscoring the prognostic value of viability assessment. The HEART trial (The Heart Revascularization Trial) further illustrates the challenges in this research era [40]. It was prematurely terminated due to recruitment difficulties, limiting its ability to provide conclusive data. The PARR-2 trial is notable for using FDG-PET to guide revascularization decisions. It demonstrated a trend toward a lower incidence of cardiovascular events in the PET-guided group compared to the standard treatment arm [39,41]. However, a 25% non-adherence rate to PET-based recommendations likely diluted the trial’s impact. A post-hoc analysis revealed that a mismatch greater than 7% on FDG-PET scans could be associated with improved outcomes following revascularization, highlighting the importance of appropriate patient selection and adherence [42].

These trials suggest that while revascularization of viable myocardium may offer clinical benefit in selected patients, it has not consistently demonstrated a survival benefit in randomized trials. It is important to note that many of these studies had significant limitations, such as suboptimal enrollment, heterogeneous imaging protocols, and non-uniform viability assessment criteria.

While large-scale trials have not conclusively demonstrated the broad benefits of viability assessment, some studies still highlight its value in select patient groups. The VIAMI trial showed that patients with recent myocardial infarction and viable myocardium detected using dobutamine stress echocardiography experienced fewer episodes of unstable angina within six months after revascularization [43]. Similarly, the ongoing CARISMA CTO trial highlights the importance of viability assessment in patients with chronic total occlusions—a population often burdened with multiple comorbidities and higher procedural risks. Invasive revascularization in these high-risk patients should be carefully reserved for those with evidence of viable myocardium to maximize benefit and minimize unnecessary harm [44]. Moreover, viability testing may have an important role in patients with ischemic cardiomyopathy and reduced ejection fraction, helping guide decisions about revascularization. Overall, incorporating viability assessment into clinical practice promotes a personalized, risk-balanced approach, ensuring that invasive treatments are directed toward those most likely to gain meaningful improvements [45].

The choice of imaging modality also plays a crucial role in evaluating hibernating myocardium. Each imaging modality presents distinct advantages and limitations that must be carefully considered in the context of individual patient characteristics and clinical scenarios.

Patient-specific factors should guide the selection of imaging techniques. PET and MRI offer more precise insights into myocardial metabolism and scar burden, respectively, while CT imaging might be preferable for claustrophobic patients. While safe and radiation-free, dobutamine echocardiography suffers from low sensitivity and higher inter-observer variability, while modalities involving radiation exposure, such as CT and PET, raise concerns when repeated imaging is necessary. A large-scale meta-analysis comparing various imaging modalities highlighted the superior sensitivity of PET (82Rb-18F-FDG) and 201 Tl rest-redistribution imaging. It also emphasizes the high specificity of dobutamine echocardiography in assessing viability. Despite its sensitivity, metabolic imaging does not always correlate with functional recovery due to its limited ability to assess structural integrity. For example, a study by Pagano et al. found that among patients with positive FDG-PET results for viable myocardium, only those with intact myofibrillar structure demonstrated an inotropic response during dobutamine stress testing, reinforcing the necessity of integrating both structural and metabolic assessment to guide treatments [46].

Following coronary artery bypass grafting (CABG) or percutaneous coronary interventions (PCI), patients may continue to experience myocardial ischemia due to progression of native coronary artery disease, incomplete revascularization, or even graft failure [36]. In this context, viability assessment plays a crucial role in guiding further management, especially when considering repeat revascularization. Imaging modalities such as cardiac MRI (CMR), positron emission tomography (PET), single-photon emission computed tomography (SPECT), and dobutamine stress echocardiography (DSE) are commonly used to differentiate viable but dysfunctional myocardium (hibernating or stunned) from non-viable scar tissue. CMR with late gadolinium enhancement offers high spatial resolution and is considered the gold standard for detecting myocardial scar [47]. PET, particularly using FDG, provides high sensitivity for detecting viable myocardium based on preserved glucose metabolism, even in areas with severely reduced perfusion [41]. SPECT, although more widely available, generally has lower sensitivity and specificity in this setting—particularly in patients with multivessel disease or prior CABG—due to factors such as attenuation artifacts, limited spatial resolution, and impaired tracer uptake in chronically ischemic but viable tissue. DSE assesses contractile reserve in hypokinetic segments and might be useful when MRI or PET are unavailable [48]. Identifying viable myocardium in post-CABG or post-PCI patients helps predict functional recovery and supports decisions regarding the potential benefit of revascularization versus medical therapy alone.

In conclusion, although advanced imaging techniques have greatly enhanced our ability to assess myocardial viability, their translation into improved survival through revascularization remains inconclusive. Each imaging modality has its strengths and limitations, impacted by sensitivity, specificity, and patient characteristics, which must be thoroughly considered in clinical decision-making. Integrating functional and structural imaging—potentially augmented by AI—offers a more comprehensive approach to identifying patients most likely to benefit from revascularization. The lack of standardized viability thresholds and non-uniform adherence to imaging-guided treatment strategies in past trials highlights the urgent need for refined protocols. Future research must prioritize prospective, multicenter studies with advanced imaging technologies, rigorous methodology, and clinically meaningful endpoints to optimize revascularization strategies and improve outcomes for patients with ischemic cardiomyopathy.

## 8. Future Directions

Advancing the clinical application of imaging techniques for assessing myocardial viability requires targeted efforts across several key domains. Central to this progress is enhanced clinical trial design, standardization, and innovation of imaging modalities. Large-scale clinical trials evaluating the role of viability-guided revascularization have historically been constrained by poor enrollment and the heterogeneity of imaging techniques. To generate meaningful data, future trials should be adequately powered and employ standardized multimodality imaging protocols. Current imaging techniques suffer from variability that limits their clinical utility and comparability across cardiac centers. Off-label modalities such as speckle tracking echocardiography show promise for viability assessment but require further validation through large-scale controlled trials to confirm their prognostic value. Similarly, standardizing imaging protocols, such as timing in delayed enhancement multi-slice computed tomography, is essential for consistency and reproducibility. Adopting low radiation dose protocols must be prioritized to reduce patient exposure, especially given the repeated imaging often required in longitudinal viability assessments.

Integrating artificial intelligence (AI) and machine learning into cardiac imaging holds significant potential. These technologies can help automate image interpretation, reduce inter-observer variability, and identify subtle patterns of hibernating myocardium. For example, Virtual Native Enhancement (VNE) technology has shown potential for replicating the diagnostic accuracy of LGE CMR without the need for contrast agents. AI-driven models can also enhance patient selection, predict response to revascularization, and personalize treatment based on imaging phenotypes and individual patient characteristics [49].

Cost-effectiveness analysis is critical to understanding the value of advanced and multimodality imaging in guiding treatment and predicting outcomes. These studies could support evidence-based policy decisions and ensure sufficient resource allocation. Furthermore, developing and adapting safer and more easily accessible imaging options, such as non-contrast cardiac MRI, will be crucial for broadening availability, especially in under-resourced settings.

## 9. Conclusions

While hibernating myocardium holds promise for advancing cardiovascular care, its clinical application remains fraught with challenges. The choice of imaging modality, the integration of metabolic and structural evaluations, and patient-specific considerations all play pivotal roles in optimizing diagnosis and treatment outcomes. (Table 2) Addressing these challenges requires the development of standardized protocols and executing robust, well-designed clinical trials with long-term follow-up. By bridging the gaps in current evidence and practice, clinicians can better harness the potential of revascularization to improve patient outcomes in cases of underperforming yet viable myocardium.

## Figures and Tables

**Table 1:** Myocardial response to low and high dose dobutamine with clinical interpretation.

Myocardium Type	Low-Dose Dobutamine Response	High-Dose Dobutamine Response	Clinical Interpretation
**Normal Myocardium**	Increased contractility	No contractility or further increase in contractility	Healthy tissue with adequate contractile reserve
**Viable Myocardium**	Improved contractility	No contractility	Hibernating/stunned myocardium with contractile reserve, potential for recovery post revascularization
**Non-viable Myocardium**	No contractility	No contractility	Necrotic tissue (no contractile reserve)

**Table 2:** Comparative analysis of imaging modalities in myocardial viability assessment.

Feature	CMR	PET	SPECT	DSE
Mechanism	Scar/fibrosis (LGE), contractile reserve	Metabolism (FDG), perfusion	Perfusion	Contractile reserve
Sensitivity	High	Very high	Moderate	Moderate
Specificity	High	High	Moderate	Moderate to high
Spatial Resolution	Excellent (1–2 mm)	Moderate (4–5 mm)	Low (10–15 mm)	Moderate
Radiation	None	Yes	Yes	None
Availability	Moderate	Limited	Widely available	Widely available
Cost	High	High	Moderate	Low
Time	Moderate (~45–60 min)	Long (60–90 min)	Long (~60–120 min for Tc-99m; longer for Thallium-201)	Short (~30 min)
Prognostic Value	Strong	Strong	Moderate	Moderate
Limitations	Contraindicated in devices, breath-hold needed	Limited access, expensive	Poor resolution, attenuation artifacts	Operator-dependent, suboptimal in poor windows

Central Illustration.

## Data Availability

No new data were generated or analyzed in the course of this review. All information is derived from previously published sources.
